# The Pathways for Layered Double Hydroxide Nanoparticles to Enhance Antigen (Cross)-Presentation on Immune Cells as Adjuvants for Protein Vaccines

**DOI:** 10.3389/fphar.2018.01060

**Published:** 2018-09-20

**Authors:** Shiyu Yan, Kewei Xu, Li Li, Wenyi Gu, Barbara E. Rolfe, Zhi P. Xu

**Affiliations:** ^1^Australian Institute for Bioengineering and Nanotechnology, The University of Queensland, Brisbane, QLD, Australia; ^2^School of Medicine, The University of Queensland, Brisbane, QLD, Australia

**Keywords:** nanoparticle adjuvant, layered double hydroxides, antigen presentation cells, dendritic cell maturation, cross-presentation, immune responses

## Abstract

Nanoparticles (NPs) are intensively investigated as adjuvants in new generation vaccines, while how these NPs promote the immune responses has not been well understood. In this research, we have tried to elucidate the possible pathways for layered double hydroxide (LDH) NPs to provoke immune responses. As previously reported, LDH NPs efficiently deliver antigens to antigen presenting cells (APCs). In this research, we have found that these internalized LDH NPs are not released by these APCs within 8 h. We have for the first time found that macrophage cells exchange the internalized LDH NPs with other surrounding ones, which may promote immune responses in an additional way. Moreover, the internalized LDH-antigen NPs significantly facilitate the maturation of immature DCs and enhance cross-presentation of epitope/MHC class I complexes on the DC surface. This research would help understand the NP adjuvant mechanism and further assist the design of new specific NPs as more efficient nano-adjuvants.

## Introduction

Various nanomaterials have been widely examined to deliver immunogens and immune stimulants as adjuvants in vaccine development (Fan and Moon, 2015; Zhang et al., 2015). For example, polystyrene delivering ovalbumin (OVA) induces systemic immune responses in sheep (Scheerlinck et al., 2006). Poly (lactic-co-glycolic acid) (PLGA) co-delivering tyrosinase-related protein 2 (TRP-2) and toll-like receptor ligand 4 (TLR4) induces cell-mediated immunity (Hamdy et al., 2008). Mesoporous silica (MS) as the antigen adjuvant also shows good adjuvant activities in HIV (Cheng et al., 2012) and porcine circovirus (Guo et al., 2012) vaccines. Calcium phosphate (Cap) adjuvanted herpes simplex vaccine shows systemic immune response in mice (He et al., 2002) and layered double hydroxide (LDH) delivering DNA vaccine shows high efficiency in transfection, and promotes immunity (Li et al., 2011; Wang et al., 2014; Williams et al., 2014). In particular, we have previously reported that LDH co-delivering OVA/TLR9 ligand CpG and Intimin β (IB) promotes potent humoral and cell-mediated immunities (Yan et al., 2014, 2018; Chen et al., 2016). However, how nanomaterials as adjuvants stimulate strong immune responses has not been well understood.

The most critical adjuvant processes include the assisted cellular uptake of antigen and subsequent antigen presentation or cross-presentation by antigen-presenting cells (APCs) (Toes et al., 1996). After subcutaneous administration of vaccine formulations, APCs are recruited to take up the nanomaterial-antigen particles, and then circulate to the local regional nodes. During this period, the nanomaterial-antigen particles are processed within the APCs to present the epitope and prime naïve lymphocytes (Slingluff, 2011). Therefore, the understanding of APCs’ cellular uptake and APCs’ antigen (cross)-presentation pathways via nanoparticles is very important in adjuvant design and development.

It has been confirmed that LDH NPs facilitate negatively charged antigens (such as BSA) to attach onto and enter the cell (Gu et al., 2015; Chen et al., 2016). As reported by Li et al. (2010) 60–65% bone marrow dendritic cells (BMDCs) took up LDH nanoparticles within 3 h. LDH is a family of anionic clay minerals, with the general formula of [M^2+^_1−x_ M^3+^_x_ (OH)_2_]^x+^[A^n−^_x/n_⋅yH_2_O]^x−^, where M^2+^ is a divalent cation, M^3+^ a trivalent cation, and A^n−^ an anion (Braterman et al., 2004). LDH has positively charged hydroxide basal layers where the trivalent cations substitute for the divalent cations, which are balanced by the hydrated anions intercalated in the interlayer space. MgAl-LDH NPs possess low toxicity and good biocompatibility, high loading of proteins and proteomic vaccines and a high capability to facilitate the cellular uptake of payloads (Xu et al., 2006c, 2008a), which may explain why LDH NPs can act as effective adjuvants to stimulate strong immune responses in vaccine development (Yan et al., 2014). However, there is no report regarding (1) whether LDH-antigen complexes facilitate maturation of APCs; (2) whether APCs that take up NPs exchange these NPs with other APCs; and (3) how antigen is cross-presented by APCs through LDH-antigen complexes.

In this study, we reinvestigated the antigen cellular uptake of LDH-dye NPs by murine macrophage cells and bone-marrow dendritic cells, and examined enhancement of presentation and cross-presentation of the model antigen OVA delivered by LDH NPs. We also employed the mimicking of antigen presentation via MHC class I pathway using LDH NPs to prime T cell activation in B3Z CD8+ T hybridoma system. Our results demonstrate the possible pathways to explain how LDH-delivered antigen significantly improves the dendritic cells maturation and enhances the antigen cross-presentation on DCs’ surface.

## Materials and Methods

### Preparation of LDH, LDH-FITC, and LDH-Congo Red (LDH-CR) NPs

Mg_2_Al(OH)_6_Cl⋅mH_2_O LDH NPs were prepared as described in previous work (Xu et al., 2006a,b). In brief, 10 mL of mixed salt solution containing MgCl_2_ 6H_2_O, (0.30 M) (Chem-Supply, 99.0–101.0%), with AlCl_3_ 6H_2_O (0.10 M) (Scharlau, 95–101%) was poured into 40 mL of NaOH (Sodium hydroxide pellets; Ajax Finechem) solution (0.15 M) under vigorous stirring. After 10 min stirring, LDH slurry was collected and washed twice with deionized water by centrifugation (SIGMA4^®^-16K Centrifuge) at 4700 rpm for 10 min. Then the slurry was manually dispersed in 40 mL of deionized water and transferred into an stainless steel autoclave with a Teflon lining (Parr Acid Digestion Vessels) for heating at 100°C for 6 h, giving rise to a homogeneously dispersed MgAl-LDH suspension.

To make LDH-FITC NPs, ¼ of manually dispersed LDH slurry was mixed with 0.5 mL of 0.025 M FITC^2−^ (fluorescein isothiocyanate; Sigma-Aldrich),, and shaken for 1 h, followed by separation and washing via centrifugation. The slurry was then manually dispersed in 10 mL of deionized water, which was similarly treated at 100°C for 6 h, yielding a well dispersed LDH-FITC NP suspension.

LDH-Congo red (LDH-CR) NPs were prepared similarly. Congo-red (0.0125 M; Sigma-Aldrich) was pre-mixed with 40 mL NaOH (0.15 M) solution before adding 10 mL of mixed salt solution containing MgCl_2_ (0.30 M) and AlCl_3_ (0.10 M). The resultant suspension was separated and the collected slurry washed twice. Finally, the slurry was dispersed in water and treated in an autoclave at 100°C for 14 h, yielding an LDH-CR NP suspension.

The particle size distribution of these LDH NP suspensions was measured with a dynamic light scattering (DLS) instrument (Nanosizer Nano ZS, MALVERN Instruments) to estimate the average hydrodynamic particle size and check the dispersion state.

### Cell Culture

RAW 264.7 macrophage cells (ATCC) were grown on 93 mm × 21 mm Petri dish in complete RPMI 1640 medium (Life Technologies Corporation, Australia) supplemented with 10% fetal bovine serum and adjusted to contain 100 μg/mL streptomycin and 100 units/mL penicillin, all from Invitrogen. Cell subcultures were made by scraping or mechanical isolation.

DC2.4 cells (kindly provided by A/Prof Mingnan Chen, University of Utah, United States) were grown in complete RPMI 1640 medium supplemented with 10% fetal bovine serum and adjusted to contain 1% L-glutamine, streptomycin and penicillin, all from Invitrogen.

Bone marrow dendritic cells were generated according a previous publication (Lutz et al., 1999). All animal studies were performed with adherence to the guidelines of the Animal Ethics Committee of The University of Queensland. Femurs and tibias were obtained from 6 to 8 weeks C57BL/6 female mice. Bone marrow was mashed into the single cell suspension via a 70 μm cell strainer. On the first day, 2 × 10^6^ BM leukocytes were seeded in each Petri dish in 10 mL complete RPMI medium with 10% fetal bovine serum and 0.05 mM of 2-mercaptoethanol and adjusted to contain 1% L-glutamine, streptomycin, and penicillin. In addition, 200 ng recombinant murine granulocyte macrophage colony stimulating factor (rmGM-CSF; Sigma-Aldrich) was added as supplement. At day 3, another 200 ng rmGM-CSF in 10 mL medium was added into the dish. At days 6 and 8, half of cell supernatant was collected, and after centrifugation, cells were resuspended into 10 mL fresh medium with 200 ng rmGM-CSF, and then added into original Petri dish.

### Cellular Uptake of LDH NPs

After subculture of RAW 264.7 macrophage cells, 35 mm × 10 mm Nunclon cell culture dishes were used for cell growth at the density of 5 × 10^5^ cells/mL for overnight. Then, 5 or 25 μg/mL LDH-FITC NP suspension was added into these dishes for cellular uptake. Controls were added with the same volume of PBS. Cells were cultured at 37°C in a 5% CO_2_ incubator and then collected at the time point of 0.5, 1, 2, 4, or 8 h. The collected cells were washed, and fixed in 4% Paraformaldehyde (PFA; Sigma-Aldrich) solution for FACS analysis (FCM, BD Accuri^TM^ C6, BD Biosciences, San Jose, CA, United States).

Similarly, freshly obtained BMDCs were cultured in 6-well plates at the density of 1 × 10^6^ DC/well in 1.5 mL medium containing 50 μg/mL LDH-FITC NPs. After incubation for 0.5, 1, 2, 4, or 8 h at 37°C, BMDCs were collected for FACS analysis to determine the uptake kinetics. For dose-dependent uptake assay, BMDCs were cultured in 1.5 mL medium containing 10, 20, 50, 100, and 200 μg/mL of LDH-FITC NPs for 2 h at 37°C, and then collected for uptake quantification using FACS. Similarly, the cellular uptake kinetics of LDH-CR NPs were also examined.

### Cellular Exocytosis of Internalized LDH NPs

To analyze the release of internalized LDH-FITC NPs by the cells, 25 μg/ml LDH-FITC NP suspension was added into the dishes for uptake by macrophages for 2 h. Then, the cells were collected and washed with PBS to remove free LDH NPs, followed by further culture in fresh medium. The cells were finally collected and washed with PBS at the time point of 0, 0.5, 1, 2, 4, or 8 h post incubation, and fixed in 4% PFA solution for FACS analysis.

### Exchange of Internalized LDH NPs Between Macrophages

Intercellular exchange of LDH NPs between macrophage cells was examined by co-culturing two individually labeled macrophage populations. In brief, 25 μg/mL of LDH-FITC and LDH-CR NPs were separately added into RAW 264.7 macrophage cell suspensions for cellular uptake for 2 h, yielding two cell populations (each labeled with a specific dye), i.e., MΦ_LDH–FITC_ and MΦ_LDH–CR_. Two cell populations were then mixed at the equal cell number and then co-cultured for 4 h in fresh medium. The co-cultured cells were collected and fixed for FACS analysis, and cell images were taken using a Carl Zeiss LSM 510 confocal laser-scanning microscope (CLSM, Carl Zeiss MicroImaging GmbH, Germany). For comparison, two cell populations, i.e., MΦ_LDH–FITC_ and MΦ_LDH–CR_, were also cultured for 4 h separately in fresh medium and analyzed.

### BMDC Maturation Induced by LDH-OVA NPs

At day 7 or 8, BMDCs were cultured in ultra-low attachment plates and pulsed with OVA (albumin from hen egg white, lyophilized powder, ≥98%, Grade VI; Sigma-Aldrich) or the equal amount of OVA in complex with LDH in RPMI 1640 medium (without GM-CSF) for 16 h. Cells were then harvested and washed, and stained with Alexa Fluor^®^ 488 anti-mouse I-A/I-E Antibody (Clone 2G9; BioLegend) to determine dendritic cell maturation. Here LDH NPs with OVA were made by mixing them at the mass ratio of 2:1, at the concentration of 200 and 100 μg/mL, respectively.

### SIINFEKL-Antigen Presentation in DC2.4 Cells Promoted by LDH NPs

After cell internalization, OVA antigen would be enzymatically degraded into the functional epitopes. OVA H-2Kb-restricted CTL epitope (OVA_257−264_, SIINFEKL) would interact with MHC class I complexes, leading to the presentation of the MHC class I-functional epitope (like SIINFEKL) complex on the surface of DCs. To do this assay, DC 2.4 cells were cultured in 96 well cell culture plates, and pulsed with OVA in complex with LDH in RPMI 1640 medium (without GM-CSF) for 16 h. Then cells were harvested and washed, and stained with APC or PE anti-mouse H-2Kb of MHC class I bound to SIINFEKL antibody (Clone 25-D1.16; BioLegend) to determine the degree of epitope presentation (SIINFEKL/H-2Kb complexes) on DC2.4 cell surface.

### B3Z CD8+ T Hybridoma Cell Activation

T cell priming can also indicate SIINFEKL epitope presented on the murine Kb MHC class I molecules (Karttunen et al., 1992). B3Z cell (kindly provided by A/Prof Mingnan Chen, University of Utah, United States), a CD8+ T-cell hybridoma, induces β-galactosidase (β-gal) production through T cell receptor interaction with SIINFEKL/H-2Kb complexes. To do this assay, DC 2.4 cells were cultured in 96 well plates at a density of 1 × 10^5^ cells/mL, and were pulsed with OVA or LDH-OVA at a designed concentration for 16 h. After washing with PBS, the same number of B3Z cells were added to DC 2.4 cells. After 24 h co-culture, cells were washed and incubated with lysis buffer and chlorophenol red β-galactoside for 4 h. After stopping the reaction by EDTA and glycine, the OD value of the buffer was measured at 570 nm with that at 635 nm as the reference, in order to measure the activation degree of B3Z cells, which also reflects the cross-presentation amount of SIINFEKL/H-2K^b^ complexes on DC 2.4 cells.

### Statistical Analysis

Data presented as mean ± standard error of the mean (SEM) were analyzed by one-way analysis of variance (ANOVA), followed by multiple comparisons using Tukey’s test within GraphPad Prism software. A *p*-value <0.05 was considered statistically significant. ^∗^*p* < 0.05; ^∗∗^*p* < 0.01; ^∗∗∗^*p* < 0.001; and ^∗∗∗∗^*p* < 0.0001.

## Results

### Physicochemical Features of LDH-FITC and LDH-CR NPs

**FIGURE 1:**
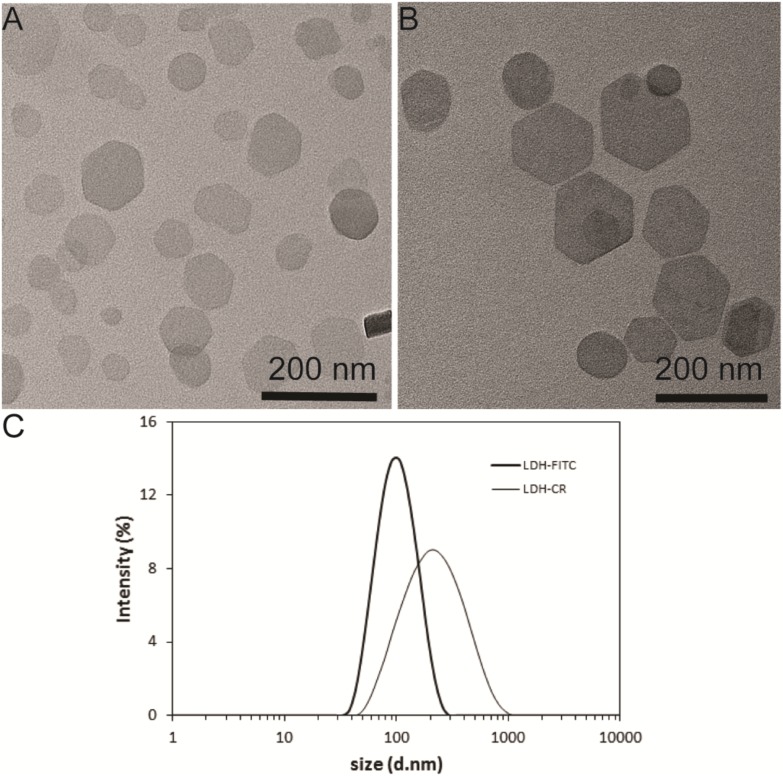
Layered double hydroxide (LDH) NP Physicochemical Feautres. TEM image of LDH-FITC **(A)** and LDH-CR **(B)** NPs; and size distribution by intensity for LDH-FITC and LDH-CR NPs **(C)** in aqueous solution.

Both LDH-FITC and LDH-CR NPs were well dispersed in aqueous suspensions, showing a moderate particle size distribution (**Figures 1A–C**). The equivalent mean hydrodynamic diameter for LDH-FITC and LDH-CR was 106 and 250 nm with the polydispersity index (PDI) of 0.132 and 0.255, respectively. Most LDH-FITC NPs were distributed within a range of 40–220 nm, while LDH-CR NPs were in 60–800 nm. The larger LDH-CR NPs may result from the longer heating time in the autoclave and the slight aggregation due to the higher CR loading. The estimated FITC was 10% of the anion exchange capacity and CR was ∼20%. The higher CR loading may also facilitate the LDH-CR crystallite growth at a relatively quicker rate than the lower FITC loading (**Figures 1A,B**; Xu and Braterman, 2003). In addition, FTIR spectra and XRD patterns confirm the layered structure of LDH-FITC and LDH-CR (**Supplementary Figure S1**), with Cl^−^ as the most abundant anion in the LDH interlayer.

Interestingly, when LDH-FITC and LDH-CR NP suspensions were mixed with culture medium separately, the average hydrodynamic particle size was increased by about 2 times (**Supplementary Figure S2**), suggesting slight aggregation caused by serum proteins through the bridging effect, as reported previously in our group (Gu et al., 2015). This slight aggregation does not severely affect the cellular uptake by immune cells, as presented shortly.

### Immune Cell’s Uptake Kinetics

**FIGURE 2:**
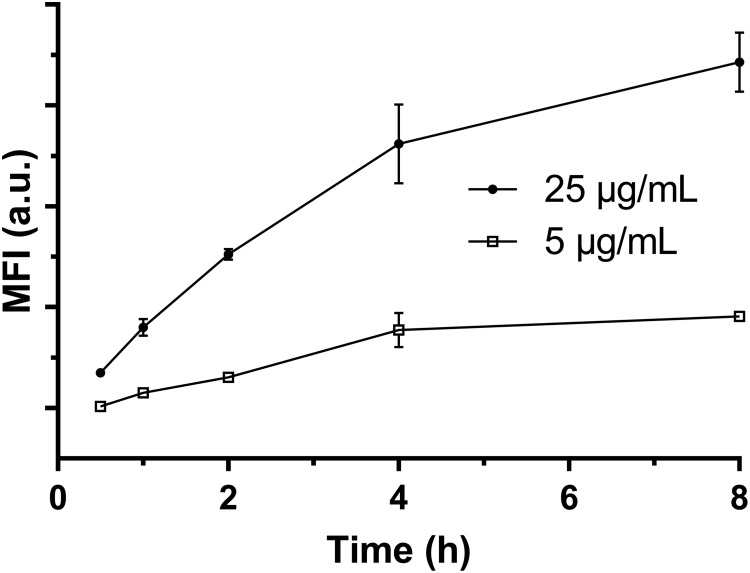
The mean fluorescent intensity (MFI) of positive viable RAW 264.7 cells after uptake of LDH-FITC NPs at 37°C in a 5% CO_2_ incubator.

The uptake kinetics of LDH-FITC NPs by immune cells (macrophages and DCs) was quantified by measuring the fluorescence intensity of each cell using the flow cytometry. As shown in **Figure 2** for macrophage uptake, the mean fluorescence intensity (MFI) was increased with the incubation time from 0.5 to 8 h at the LDH-FITC concentration of 5 and 25 μg/ml, respectively, indicating the cellular uptake is time-dependent. Interestingly, at both LDH-FITC doses, MFI increase was relatively quicker in the first 4 h than in the subsequent 4 h, as previously observed for the uptake of many other cells (Xu et al., 2008b; Oh et al., 2009; Wong et al., 2010).

Relatively, the uptake amount (MFI) at the low dose of LDH-FITC NPs (5 μg/ml) is much smaller than that at the higher dose (25 μg/ml) at all incubation time points, reflecting the cellular uptake is dose-dependent. In particular, FITC-positive cells reached 85–95% just after incubation for 1–2 h at the higher dose, i.e., almost all cells took up an enough amount of LDH-FITC in 1–2 h (**Supplementary Figure S3**) to distinguish themselves from un-treated cells. This thus indicates that the uptake of LDH-FITC NPs by macrophage cells is very rapid, and in consistence with our previous findings for other cells (Xu et al., 2008b; Musumeci et al., 2010). Similarly, LDH-CR NPs were also quickly taken up by macrophage cells (**Supplementary Figure S4**; Oh et al., 2009). The quick cellular uptake of LDH NPs can be largely attributed to the quick endosomal escape of LDH NPs during endocytosis, as reported previously (Ladewig et al., 2010; Gu et al., 2011).

As further shown in **Supplementary Figure S5**, the freshly obtained BMDCs took up LDH-FITC NPs also quickly, in a dose- and time-dependent way, as reported previously for BMDCs (Li et al., 2010) and other mammalian cells (Xu et al., 2008b; Oh et al., 2009).

### No Exocytosis of Internalized LDH NPs by Macrophage Cells

Our results indicate that there were ∼90% FITC-positive macrophage cells after culture for 2 h at the LDH-FITC dose of 25 μg/ml (**Supplementary Figure S3**), as also shown as the point at 0 h in **Supplementary Figure S6**. Thus, these cells took up an essential amount of LDH-FITC NPs. After replacement of LDH-FITC containing medium with fresh medium, these LDH-FITC-positive cells were further cultured to examine whether they release the internalized LDH-FITC NPs.

**FIGURE 3:**
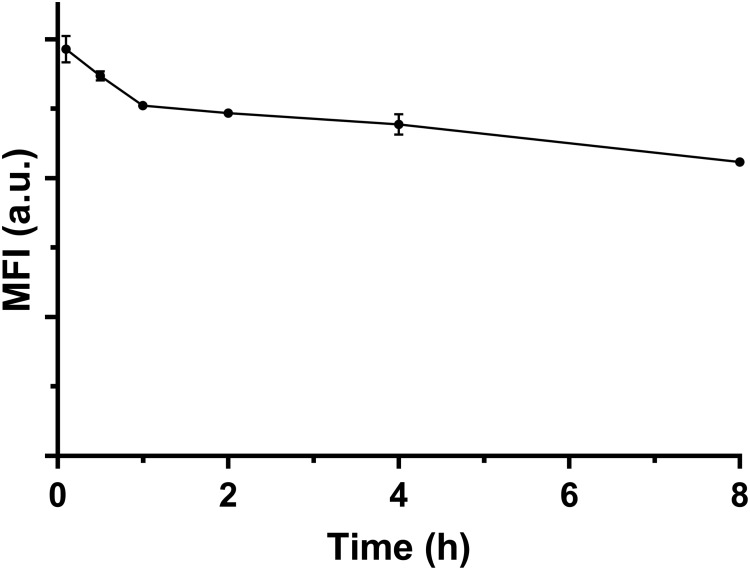
The MFI of FITC-positive viable cells changing with the incubation time in fresh medium at 37°C.

As shown in **Figure 3**, the relative MFI was reduced from 100 to 72% (28% reduction) after 8 h incubation in fresh culture medium. In particular, the relative MFI decreased from 100 to 86% (14% reduction) in the first 1 h, much more quickly than in the subsequent 7 h (14%). There are a few possible factors that contribute to the reduction of LDH-FITC NPs in each cell. The first factor is cell division. The total cell number may increase by ∼30% through division after 8 h incubation supposing that the cycle time of RAW 264.7 cells is 15–20 h. We believe that the cell division would largely explain the MFI reduction during this 8 h. The second factor is the release of the fluorescent tag (FITC) from the LDH interlayer, which may be degraded by the cell or diffuse out of the cell. If FITC release takes place in later endosome, the free FITC may also be quenched in the low pH environment. FITC release and quench may be responsible for the quick MFI reduction in the first 1 h just after cells were treated. The third factor is cellular exocytosis (release), which seems to contribute little to the MFI reduction.

Based on this test and analysis, we may conclude that macrophage cells do not obviously exocytosize the internalized LDH NPs, but keep them within the cells and passage to the next generation. As reported previously, iron oxide nanoparticles (IONPs) internalized by cells are retained within the cells and passaged to the cells in subsequent 3–4 generations (Gu et al., 2005).

### LDH NP Intercellular Exchange Between Macrophage Cells

**FIGURE 4:**
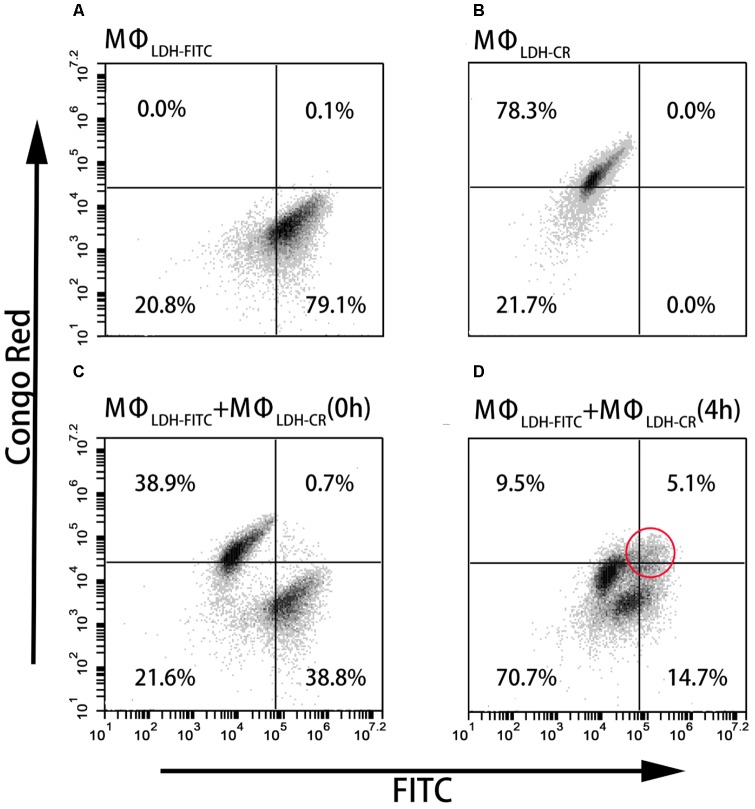
FACS dot plots showing the cell staining. **(A–D)** The dot plots showing Flow cytometric analysis for intercellular exchange of LDH NP by macrophages. **(A)** LDH-FITC macrophages (MΦ_LDH–FITC_); **(B)** LDH-CR macrophages (MΦ_LDH–CR_); **(C)** Quick mixture of LDH-FITC macrophages and LDH-CR macrophages [MΦ_LDH–FITC_ + MΦ_LDH–CR_ (0 h)]; and **(D)** 4-h mixture of LDH-FITC macrophages and LDH-CR macrophages [MΦ_LDH–FITC_ + MΦ_LDH–CR_ (4 h)].

As shown in **Figures 4A,B**, and **Supplementary Table S1**, the fluorescence intensity of LDH-FITC and LDH-CR NP-treated cells (MΦ_LDH–FITC_ and MΦ_LDH–CR_) was increased from 7,300 (MΦ_control_) to 176,000 (MΦ_LDH–FITC_) and from 2,400 (MΦ_control_) to 50,000 (MΦ_LDH–CR_) after 2-h uptake and 4-h post-incubation (**Supplementary Table S1**), with ∼80% cells being fluorescence positive (**Table 1**). When MΦ_LDH–FITC_ and MΦ_LDH–CR_ cells were mixed in the equal number and the fluorescence intensity was quickly measured. The cytometry profile was their simple combination (**Figures 4A–C**), i.e., half of their individual positive cell percentage, i.e., 39% for each population [**Table 1**, MΦ_LDH–FITC_ + MΦ_LDH–CR_ (0 h) and **Figure 4C**], without obvious change for the intensity of these two cell populations (**Supplementary Table S1**).

**Table 1:** Positive macrophage cell (MΦ) percentage labeled with FITC and CR.

Percentage (%)	MΦ_control_	MΦ_LDH–FITC_	MΦ_LDH–CR_	MΦ_LDH–FITC_ + MΦ_LDH–CR_ (0 h)	MΦ_LDH–FITC_ + MΦ_LDH–CR_ (4 h)
FITC^+^ cell	0	79.1	0	38.8	14.7
CR^+^ cell	0	0	78.3	38.9	9.5
FITC^+^ + CR^+^ cell	0	0.1	0	0.7	5.1

After 4 h incubation of the mixed cells, two populations moved into the cross to close each other (**Figure 4D** and **Supplementary Figure S7**). For example, the FITC intensity of two cell populations [MΦ_LDH–FITC_ + MΦ_LDH–CR_ (4 h)] was 26,800/64,200, in sharp contrast to 12,400/173,000 [MΦ_LDH–FITC_ + MΦ_LDH–CR_ (0 h)] (**Supplementary Table S1**). Similarly, the CR intensity was 4,500/18,700 vs. 3,400/49,700, respectively (**Supplementary Table S1**). Very remarkably, there were 5.1% cells being both FITC-positive and CR-positive (**Figure 4D**, indicated with the red circle), while the percentage of only FITC-positive and only CR-positive cells was significantly reduced to 14.7 and 9.5%, respectively [**Table 1**, MΦ_LDH–FITC_ + MΦ_LDH–CR_ (4 h)]. The histograms indicate that MΦ_LDH–FITC_ + MΦ_LDH–CR_ (4 h) cells obviously shift in both FITC and Congo red channels (**Supplementary Figure S7**), and confirmed that some cells in MΦ_LDH–FITC_ + MΦ_LDH–CR_ (4 h) population contained both LDH-FITC and LDH-CR NPs, clearly showing that macrophage cells exchange the LDH NPs with each other. The nanoparticle exchange may occur via the possible mechanisms for the transfer of antigens between APCs, such as synapse (Mittelbrunn and Sanchez-Madrid, 2012), via tunneling nanotubes (TNT) (Domhan et al., 2011), or through gap junctions (Yewdell and Dolan, 2011).

**FIGURE 5:**
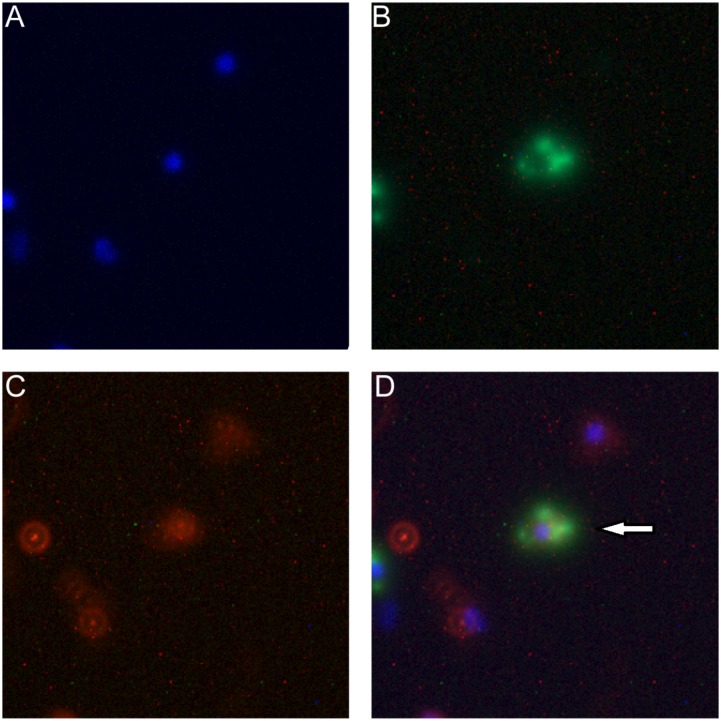
Fluorescence microscope images of macrophage cells being both FITC-positive (green) and LDH-CR (4 h). MΦ_LDH-FITC_ + MΦ_LDH-CR_- positive (red) after 4 h incubation [M Channels: **(A)** Nuclei (blue); **(B)** LDH-FITC; **(C)** LDH-CR; **(D)** Merged]. The arrow pointing the cell with both LDH-FITC and LDH-CR NPs inside.

This exchange has been also captured in the fluorescence image. As shown in **Figure 5D**, the arrow indicates that the macrophage cell has both LDH-FITC and LDH-CR NPs inside. These data thus reveal that the macrophage cells exchange the internalized NPs with their neighbors, which appeared to occur even in suspension when the two populations of cells were just mixed and contacted within a minute, as there were 0.7% cells being already both FITC-positive and CR-positive [**Table 1**, MΦ_LDH–FITC_ + MΦ_LDH–CR_ (0 h)].

### BMDC Maturation Promoted by LDH-OVA

High expression of MHC class II complexes on the DC surface, i.e., DC maturation, is very critical for generation of a high level of antigen-specific antibody (Kukutsch et al., 2000). To demonstrate the maturation effect of LDH-OVA vaccine formulation, BMDCs cultured at day 7 or 8 were collected as the target DCs. LDH-OVA was formulated at the LDH:OVA mass ratio of 2:1, and BMDCs were then exposed to culture medium containing this LDH-OVA formulation for 16 h. The I-A/I-E antibody was used to distinguish two kinds of DC subpopulations, i.e., MHC II high and MHC II low (**Supplementary Figure S8**), which are representative for mature and immature DCs according to the previous study (Kukutsch et al., 2000).

**FIGURE 6:**
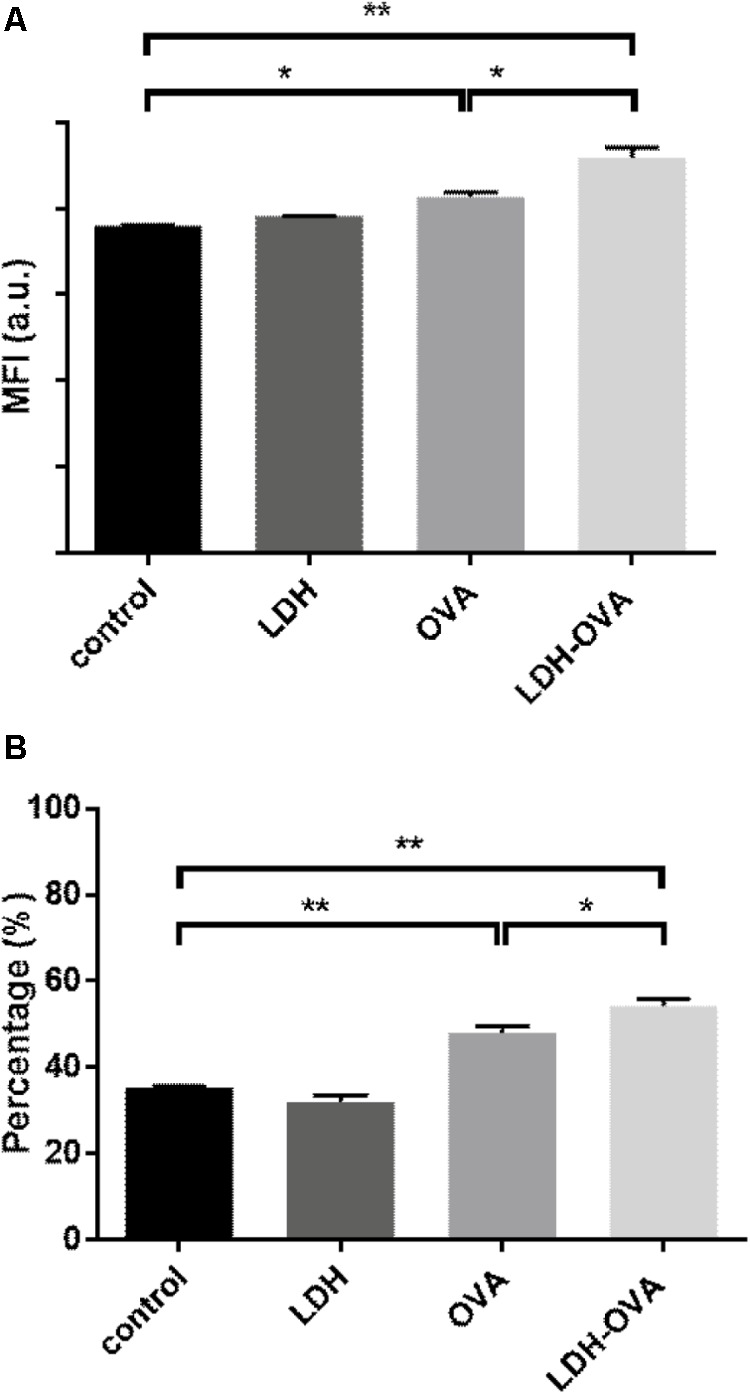
Layered double hydroxide-ovalbumin (LDH-OVA) induces BMDC maturation. The MFI **(A)** and percentage **(B)** of MHC class II high population. The data shown are representative for three independent experiments. Data are the mean ± SEM. ^∗^*p* < 0.05; ^∗∗^*p* < 0.01 (one-way ANOVA, with a Tukey’s multiple comparison test).

Our data show that there was a significant increase in terms of mature DCs when DCs were stimulated by the LDH-OVA formulation. As shown in **Figure 6A**, the MFI of MHC II high DC population treated with LDH-OVA was significantly higher than that of the blank control and OVA only-activated DC group. Consistently, the mature DC was up to 54.2% when LDH-OVA was used to stimulate DCs, significantly higher than the control group (35.4%) and OVA-stimulated group (48.0%) (**Figure 6B**). Thus LDH NPs significantly promote the maturation of DCs, as reported previously (Li et al., 2010).

### Enhanced Antigen Cross-Presentation Promoted by LDH NPs

As previously presented, blank LDH NPs are readily taken up by macrophage cells and DCs, which can be used to carry the target antigens and facilitate their cellular uptake, such as BSA (Chen et al., 2016) and OVA. After internalization, OVA antigen is probably dissociated from LDH-OVA particles either in later endosome or cytoplasm, and then enzymatically degraded into the functional epitope. This epitope interacts with MHC class I complexes, leading to the presentation of the MHC class I-functional epitope (like SIINFEKL) complexes on the surface of DCs.

**FIGURE 7:**
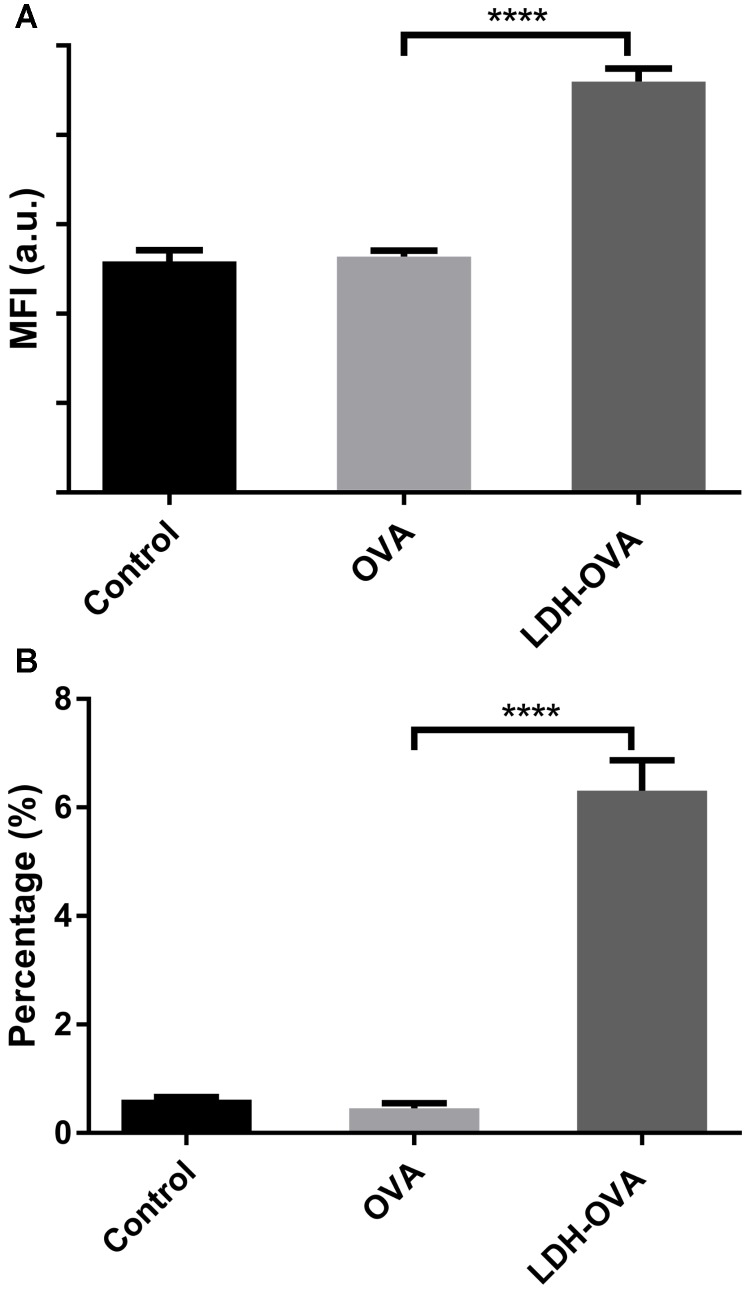
Enhanced presentation of SIINFEKL/MHC I complexes in DC 2.4 cells by LDH-facilitated OVA delivery. The cell MFI **(A)** and the percentage **(B)** of cells expressing SIINFEKL/H-2Kb complexes. Data are the mean ± SEM. ^∗∗∗∗^*p* < 0.0001 (one-way ANOVA, with a Tukey’s multiple comparison test).

In this research, 25-D1.16 antibody was used to specifically bind with the complex (SIINFEKL/H-2K^b^) to confirm and quantify the antigen cross-presentation through the MHC class I pathway, which is necessary for inducing the formation of anti-tumor CTL CD8+ T cells (Burgdorf et al., 2007). As shown in **Figure 7A**, LDH-OVA vaccine significantly enhanced the presentation of SIINFEKL/MHC I complexes on the surface of DC 2.4 in terms of the MFI, with up to 6.3% of DC 2.4 presenting complexes, in sharp contrast with nearly no antigen presented in DC 2.4 cells treated with OVA only (0.45%) and control medium (0.62%) (**Figure 7B**). The higher antigen-complex presentation on the DC surface may be largely attributed to the promoted cellular uptake and moreover, the enhanced subsequent processes, such as enzymatic degradation of OVA to epitope with the help of LDH NPs, as well as formation of MHC I-epitope complexes through the cytosolic pathway with endoplasmic reticulum (ER) or phagosomal loading (Joffre et al., 2012). Moreover, as shown in **Supplementary Figure S9**, LDH-SIINFEKL vaccine resulted in high SIINFEKL presentation by DCs which is comparable with the presentation using free SIINFEKL as the positive control. Free SIINFEKL peptide is well known to be readily loaded onto MHC class I after exogenous loading/incubation with DC’s or APCs (Cho et al., 2016).

### Enhanced T Cell Priming by LDH-OVA-Treated DCs

**FIGURE 8:**
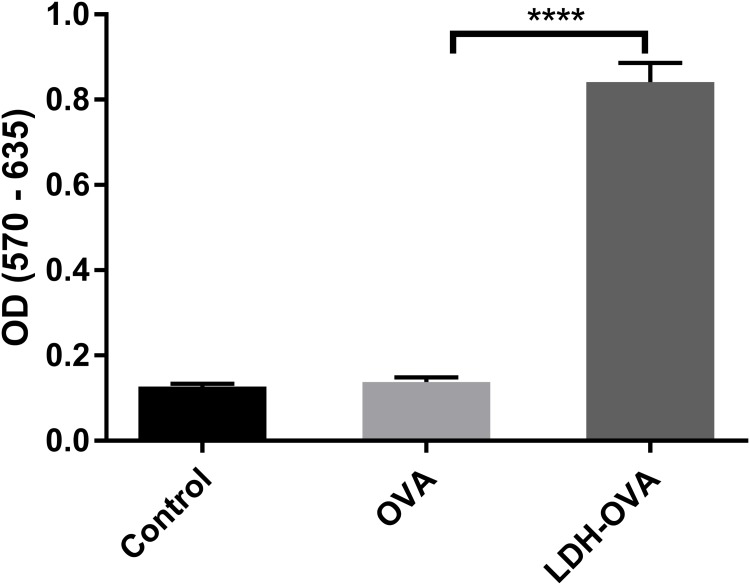
Activation of B3Z cells after co-incubation with LDH-OVA pulsed DC 2.4. Data are the mean ± SEM. ^∗∗∗∗^*p* < 0.0001 (one-way ANOVA, with a Tukey’s multiple comparison test).

T cell priming can also indicate whether the antigen is cross-presented on the DC surface in the form of MHC class I-epitope complexes. Thus, B3Z cell, a CD8+ T-cell hybridoma, was chosen to specifically recognize SIINFEKL epitope presented on the murine Kb MHC class I molecules (Karttunen et al., 1992) and determine the T cell priming extent. The priming of the SIINFEKL epitope to B3Z cells induces β-galactosidase (β-gal) synthesis by B3Z cells. The induced β-gal amount thus quantifies the cross-presentation of SIINFEKL/H-2K^b^ complexes on DC cells and the degree of T cell activation. In this research, an equal number of B3Z and DCs (LDH-OVA stimulated) were co-cultured for 24 h, and the OD value, i.e., the β-gal amount produced by B3Z, was measured in a plate reader. As shown in **Figure 8**, the OD value of lyzed B3Z cells that were co-cultured with LDH-OVA pulsed DCs was significantly higher than that of other two control groups, indicating that B3Z cells were significantly activated and further confirming that the OVA epitope was successfully cross-presented on DC 2.4 cells via LDH NP adjuvants.

## Discussion

Layered double hydroxide nanomaterials are reported to significantly promote the immune responses in mice model and show a high promise as effective nano-adjuvants (Li et al., 2011; Wang et al., 2014; Williams et al., 2014). In particular, our groups have demonstrated that LDH NPs are able to induce both high-level antibody and cellular immune responses for antibacterial and anti-tumor treatment (Yan et al., 2014; Chen et al., 2016). Apparently, the adjuvanticity of LDH NPs is related to the particle size and composition, the mass ratio of LDH: antigen, and the dose injected, which have been reported (Williams et al., 2014; Yan et al., 2014; Chen et al., 2016). In principle, the activity is largely determined by the effects of LDH NPs on the biological processes of immune cells, including long-term stimulation (depot effect), cellular uptake, APC maturation, antigen processes within APCs and the antigen presentation on the APC surface, as well as the activation of target T and B cells, which have been well investigated in the current research, together with our previous work (Yan et al., 2014; Chen et al., 2016).

When LDH-antigen NPs are injected subcutaneously, these NPs form a loosely aggregated lump, which then causes a so-called depot effect, i.e., long-term stimulation. For example, the lump of LDH NP-adjuvanted vaccine was found to last for ∼1 month beneath the skin, giving a higher and sustained level of specific antibody (Chen et al., 2016, 2018). Thus, the depot effect is beneficial to the long-term immune responses (Mckee et al., 2007; Henriksen-Lacey et al., 2010). Moreover, the lump recruits many inflammatory cells (Aimanianda et al., 2009; Chen et al., 2018), thus LDH-antigen NPs on the lump surface can be readily taken up by or facilitate the delivery of antigen (such as OVA) to these immune cells. The current research has further confirmed that LDH NPs are readily taken up by macrophage cells (**Figure 2**) and BMDCs (**Supplementary Figure S5**), which is also supported by previous reports (Li et al., 2010; Wang et al., 2014). As revealed elsewhere, this facilitation to cellular uptake results from the quick endosome escape (Choy et al., 2004; Xu et al., 2008b). As weakly alkaline LDH NPs are partly dissolved in the slightly acidic endosome, so the ion concentration increases and the enhanced osmotic pressure inside the endosome leads to water influx and bursts the endosome, releasing the LDH NPs into the cytoplasm (Xu et al., 2008b; Gu et al., 2011). Therefore, antigen is mostly associated with LDH NPs after endosome escape and then possibly processed to load with MHC I molecules through the cross-presentation pathway. This process is very much different from the case using polymeric nanoparticles to adjuvant antigens through lysosomal pathway (Lai et al., 2007, 2008; Fernando et al., 2010).

**FIGURE 9:**
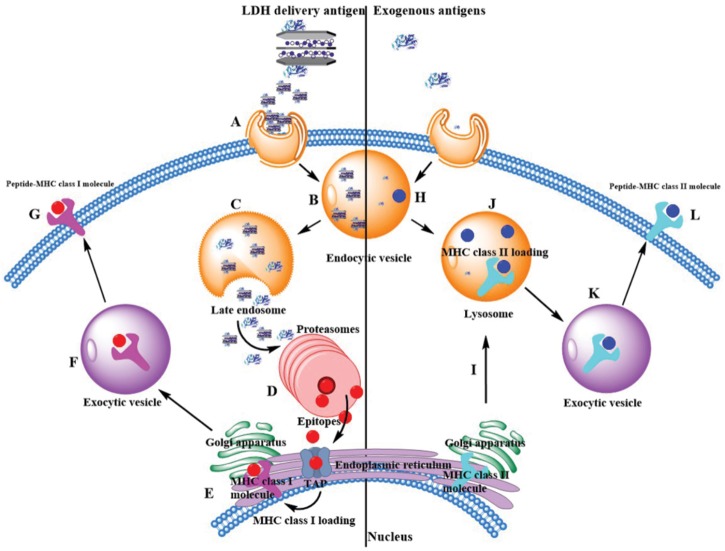
Exogenous antigen delivery mechanism through MHC II pathway by itself or through MHC I cross-presentation pathway by LDH NPs. **(Left)** LDH-based vaccine through endocytosis (Step A) is internalized into the endosome (Step B). Through the endosome escape, free antigen, and LDH-based vaccine are released into the cytoplasm (Step C), and antigens are enzymatically degraded into functional epitopes within proteasomes (Step D). With the help of transporter associated with antigen processing (TAP), epitopes are transferred into endoplasmic reticulum (ER), where they are loaded with MHC class I molecules (Step E). Afterward, these complexes are transferred to exocytic vesicle by Golgi apparatus (Step F). Finally, MHC class I complexes present these epitopes on the surface of antigen presentation cells (Step G). **(Right)** Generally exogenous antigens are intracellular internalization in endocytic vesicles, such as acidic intracellular compartments. Antigens are degraded into epitopes (Step H). Then endocytic vesicles fuse with lysosome, where epitopes are loaded with MHC class II molecules (Step J). Note that MHC class II molecules are made inside the ER and transferred to lysosome by Golgi apparatus (Step I). Finally, these epitopes are presented with MHC class II complexes on the cell surface (Step L) by exocytic vesicles (Step K).

The most potent APCs are immunologically competent dendritic cells (DCs), while their ability to regulate immunity is dependent on their maturation (Banchereau and Steinman, 1998). After the LDH-antigen vaccine is injected subcutaneously, immature DCs are recruited to the site of inflammation in peripheral tissues, and take up LDH-antigen NPs (Waeckerle-Men et al., 2004; Chen et al., 2016, 2018). In this research, we found that LDH NPs assist OVA to mature DCs by promoting significantly more MHC II complexes on the DCs’ surface (**Figure 6**), in coordination with the activation signals received from the surrounding cytokines and chemokines, costimulatory molecules and proteases (Li et al., 2010; Williams et al., 2014). On the other hand, the exogenous OVA antigens are quickly delivered to cytoplasm by LDH NPs, and processed into epitopes for complexing with MHC I molecules (cross-presentation), which is also benefited from LDH’s quick endosome escape (as schematically shown in **Figure 9**). Meanwhile, LDH-antigen NPs could also attract the proteasome and enzymatic proteases. These proteases on the LDH surface may more efficiently process the adjacent antigens into antigenic epitope. Moreover, short-chain epitope is more easily released from the LDH surface, which may quickly form more epitope-loaded MHC class II and I complexes and their subsequent (cross)-presentation. This postulated mechanism may be supported by the enhanced MHC class II high population and more SIINFEKL/MHC I complexes in LDH-OVA group (**Figures 6**, **7**), respectively. Therefore, the mature DCs have significantly enhanced presentation of antigen-loaded MHC class I complexes on the cell surface upon the stimulation of the LDH-antigen vaccine.

A more interesting issue is that immune cells (such as APCs) that take up LDH-antigen NPs may exchange these NPs with surrounding immature cells (including macrophages and DCs) at the site of injection, during the circulation in blood/lymph systems, and in the lymphoid nodes. As demonstrated in this research, macrophage cells exchanged their internalized LDH NPs with each other during *in vitro* culture (**Figures 4**, **5**). In such a way, DCs that take up LDH-antigen NPs at the injection/inflammatory site may transfer these LDH NPs to the surrounding immature DCs, thus “infect” and activate these immature DCs to mature (epitope/MHC class II presentation) and present epitope/MHC class I complexes (cross-presentation). As reported recently, cellular communication by exchanging materials may occur via synapse (Mittelbrunn and Sanchez-Madrid, 2012), tunneling nanotubes (TNT) (Domhan et al., 2011), or gap junctions (Yewdell and Dolan, 2011). Subsequently, this exchange induces a high level of specific antibody by stimulating B cells and activates more potent cytotoxic T cells for cell-mediated immune response, as reported in our previous paper (Yan et al., 2014) and this research (**Figure 8**), leading to the remarkable improvement in the immune responses.

## Conclusion

In summary, we report that APCs (such as macrophages and DCs) can take up LDH NPs efficiently, and more significantly macrophages exchange the internalized LDH NPs with surrounding ones. We also report that the internalized LDH-antigen NPs can significantly facilitate the maturation of immature DCs and enhance the antigen cross-presentation of MHC I complexes on the DC surface. The high adjuvanticity of LDH NPs may be attributed to specific properties of LDH materials, such as the weak alkalinity for endosome escape and capability of co-adsorbing enzymes on the surface for enzymatic degradation. These findings may provide some guidelines for design new adjuvants for next generation vaccines.

## Author Contributions

ZX and LL designed the current experiments in consultation with WG and BR. SY conducted most of the experiments and collected and analyzed the data. KX assisted in the experiments and data collection. SY and ZX wrote the manuscript. LL, KX, WG, and BR contributed to the revisions of the manuscript.

## Conflict of Interest Statement

The authors declare that the research was conducted in the absence of any commercial or financial relationships that could be construed as a potential conflict of interest.
