# Supplementary material for: The Pathways for Layered Double Hydroxide Nanoparticles to Enhance Antigen (Cross)-Presentation on Immune Cells as Adjuvants for Protein Vaccines
Source: Front Pharmacol. 2018 Sep 20;9:1060. doi: 10.3389/fphar.2018.01060 (PMC6158326; doi:10.3389/fphar.2018.01060)
Supplement: Supplementary file 1 [file Table_1.DOCX]

Supplementary Material (SM)

**The pathways for layered double hydroxide nanoparticles to enhance antigen (cross)-presentation on immune cells as adjuvants for protein vaccines**

Shiyu Yan, ^a^ Kewei Xu, ^b^ Li Li, ^a^ Wenyi Gu, ^a^ Barbara E. Rolfe, ^a^ and Zhi P. Xu, ^a,*^

^a^ Australian Institute for Bioengineering and Nanotechnology, The University of Queensland, Brisbane, QLD 4072, Australia.

*^b^* School of Medicine, The University of Queensland, Brisbane, QLD 4072, Australia.

*To whom correspondence should be addressed: Prof Zhi Ping Xu. Tel: 61-7-33463809. Fax: 61-7-33463973. E-mail: [gordonxu@uq.edu.au](mailto:gordonxu@uq.edu.au)

Table S1. Positive macrophage cell (MΦ) count-based peak intensity labelled with FITC and CR.

| Peak Intensity | MΦ_control_ | MΦ_LDH-FITC_ | MΦ_LDH-CR_ | MΦ_LDH-FITC_ + MΦ_LDH-CR_ (0 h) | | MΦ_LDH-FITC_ + MΦ_LDH-CR_ (4 h) | |  |
| --- | --- | --- | --- | --- | --- | --- | --- | --- |
| FITC | 7,300 | 176,000 | 8,600 | | 12,400/173,000 | | 26,800/64,000 | |
| CR | 2,400 | 3,400 | 50,000 | | 3,400/49,700 | | 4,500/18,700 | |

**(A)**

**(B)**

Figure S1. XRD pattern (A) and FTIR (B) of LDH-FITC and LDH-Congo Red.

Figure S2. Size distribution by intensity for LDH-FITC and LDH-CR in medium.

Figure S3. The time-course percentage of FITC positive viable cells after cellular uptake of LDH-FITC by RAW 264.7 incubated at 37 ^o^C.

Figure S4. The time-course of percentage of positive viable Raw 264.7 cells after uptake of LDH-Congo Red NPs (25 µg/ml) at 37 °C in a 5% CO_2_ incubator.

**(A)**

**(B)**

Figure S5. BMDC uptake of LDH-FITC. Dose- (A) and time-dependent (B) uptake kinetics.

Figure S6. The percentage of FITC-positive viable RAW 264.7 cells vs. the incubation time in fresh medium.


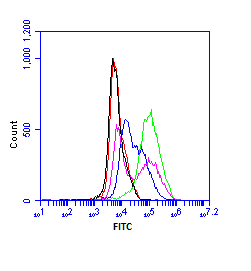

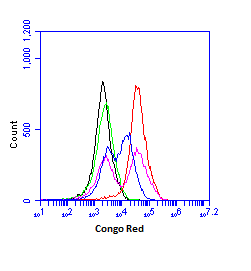


A

B

Figure S7. The histograms showing the LDH-FITC-CR macrophage distribution in FITC channel (B) and Congo Red channel (C) after 4 h co-incubation in various conditions. ■ Blank macrophages (MΦ_control_); ■ LDH-FITC macrophages (MΦ_LDH-FITC_); ■ LDH-CR macrophages (MΦ_LDH-CR_); ■ Quick mixture of LDH-FITC macrophages and LDH-CR macrophages (MΦ_LDH-FITC_+ MΦ_LDH-CR_ (0 h)); ■ 4-h mixture of LDH-FITC macrophages and LDH-CR macrophages (MΦ_LDH-FITC_+ MΦ_LDH-CR_ (4 h)).


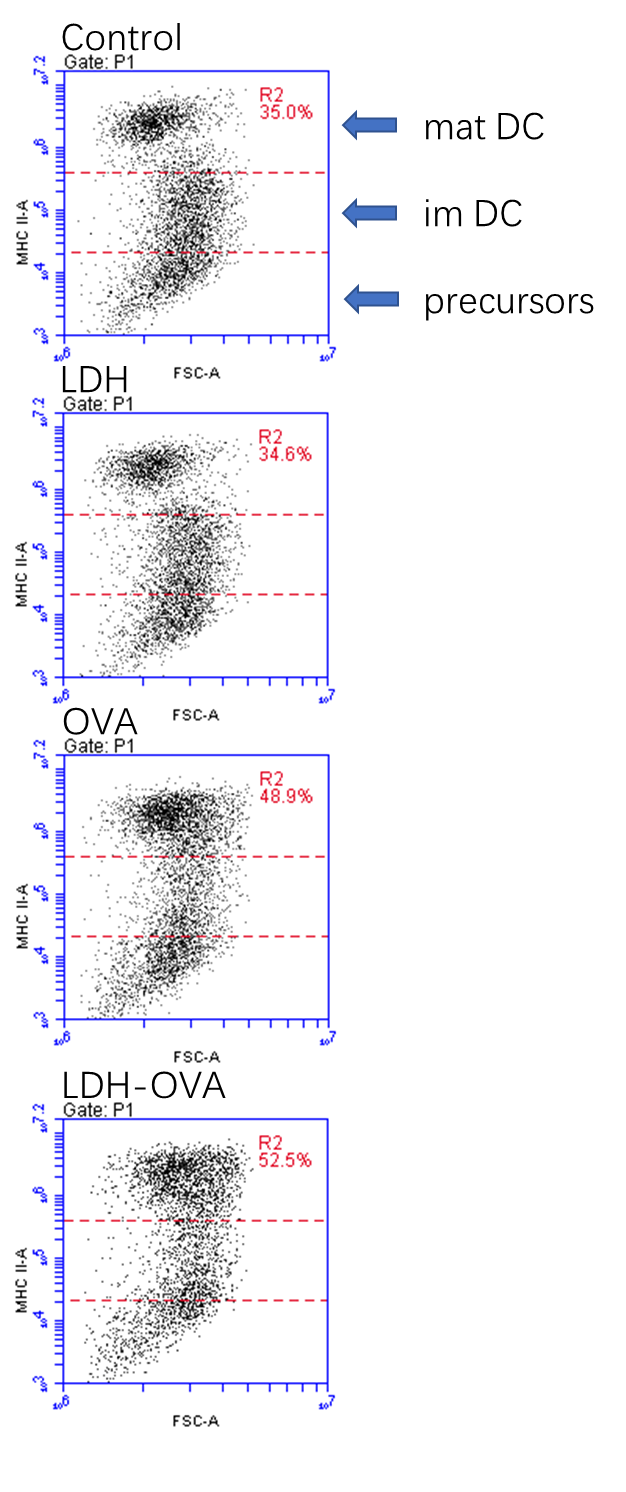


Figure S8. Dot plots and the gating strategy used to identify the change in MHC class II expression, as mature DC (MHC II^high^), immature DC (MHC II^low^) and granulocytes/precursor (MHC II^neg^).

Figure S9. ▏Presentation of SIINFEKL/MHC I complexes in DC 2.4 cells by LDH-facilitated SIINFEKL delivery. The cell MFI (A) and the percentage (B) of cells expressing SIINFEKL/H-2Kb complexes.
